# Description of a new species of beaked whale (*Berardius*) found in the North Pacific

**DOI:** 10.1038/s41598-019-46703-w

**Published:** 2019-08-30

**Authors:** Tadasu K. Yamada, Shino Kitamura, Syuiti Abe, Yuko Tajima, Ayaka Matsuda, James G. Mead, Takashi F. Matsuishi

**Affiliations:** 1 Department of Zoology, National Museum of Nature and Science, Tsukuba, Ibaraki Japan; 2 Sanriku Fisheries Research Centre, Iwate University, Kamaishi, Iwate Japan; 3 Faculty of Fisheries Sciences, Hokkaido University, Hakodate, Hokkaido Japan; 4 Division of Mammals, Smithsonian Institution, Washington, D.C. USA; 5 Global Institution for Collaborative Research and Education, Hokkaido University, Hakodate, Hokkaido Japan

**Keywords:** Taxonomy, Zoology

## Abstract

Two types of *Berardius* are recognised by local whalers in Hokkaido, Japan. The first is the ordinary Baird’s beaked whale, *B*. *bairdii*, whereas the other is much smaller and entirely black. Previous molecular phylogenetic analyses revealed that the black type is one recognisable taxonomic unit within the *Berardius* clade but is distinct from the two known *Berardius* species. To determine the characteristics of the black type, we summarised external morphology and skull osteometric data obtained from four individuals, which included three individuals from Hokkaido and one additional individual from the United States National Museum of Natural History collection. The whales differed from all of their congeners by having the following unique characters: a substantially smaller body size of physically mature individuals, proportionately shorter beak, and darker body colour. Thus, we conclude that the whales are a third *Berardius* species.

## Introduction

Beaked whales (Family Ziphiidae, Odontoceti, Cetacea) include the second largest number of species among toothed whale families. Their preference for deep ocean waters, elusive habits, and long dive capacity^1^ make beaked whales hard to see and inadequately understood. A total of 22 species are currently recognized in six genera (*Berardius*, *Hyperoodon*, *Indopacetus*, *Mesoplodon*, *Tasmacetus*, and *Ziphius*)^2^. The genus *Berardius* has two species, Baird’s beaked whale *Berardius bairdii*, found in the North Pacific and adjacent waters, and Arnoux’s beaked whale *B*. *arnuxii*, found in the Southern Ocean^3^. Besides the two nominal species, however, whalers’ observations off Hokkaido, northern Japan, have alluded to the occurrence of two groups of *Berardius*, one being slate-gray form and the other, the black form, which are smaller in body size^4,5^. Today, slate-gray form is common around Japan, which are traditionally considered as *B*. *bairdii*, but black form is rare, and no detailed morphological examinations have been conducted so far. Recent molecular phylogenetic analyses strongly suggest the black and the slate-gray forms in the North Pacific as genetically separate stocks of *Berardius*^6,7^, awaiting further work with sufficient morphological data to verify the differences between the two types of *Berardius*.

Here, we examined black type beaked whale external morphology and skull osteometric data obtained from four specimens including three from Hokkaido and one from the United States National Museum of Natural History (USNM) collection, to highlight the morphological characteristics of the black form after comparison with those of their congeners, *B*. *bairdii* and *B*. *arnuxii*. The observed unique external characters and skull osteomorphology, coupled with updated molecular phylogeny of *Berardius*, distinguish the black form as a third *Berardius* species previously unknown in cetacean taxonomy.

## Genus ***Berardius***

Before discussing the above-mentioned subject, it would be useful to summarise what is known about the genus *Berardius*. *Berardius* was established by Duvernoy in 1851^8^, who described *B*. *arnuxii* based on a specimen collected in New Zealand. The skull and mandibles of this individual are preserved in le Museum Nationalle d’Histoire Naturelle (MNHN) in Paris. Stejneger^9^ described a similar species of this genus, *B*. *bairdii* Stejneger (USNM 20992), as a northern counterpart in 1883; this description was published just a few months earlier than Malm’s^10^ description of *B*. *vegae*, which was later defined as a junior synonym of *B*. *bairdii*^11^. Both specimens were collected from Bering Island. The *B*. *bairdii* holotype includes a skull and mandibles, and the *B*. *vegae* holotype consisted of broken skull pieces. *B*. *arnuxii* and *B*. *bairdii* could be good examples of antitropical distribution^12^.

As summarised by Kasuya^5,13^, there have been extensive debates on the identities of these two species, because they are very similar except for body size and distribution. *B*. *arnuxii* is slightly smaller than *B*. *bairdii*. True^11^ pointed out several characters that are distinct between these two species. However, as the number of specimens increased, most of the characters lost systematic significance, and their validity was disputed^14,15^. Dalebout *et al*.^16^ put an end to this discussion and showed that the two species are genetically distinct and independent. However, morphological discrimination of these two species is not currently well established and we have to rely on molecular results or distribution to discriminate these two species. Ross^17^ noted that more thorough morphological investigations are needed to distinguish *B*. *bairdii* and *B*. *arnuxii*.

*Berardius* skulls are the least asymmetrical and sexually dimorphic among genera of the family Ziphiidae; only the body length of females is slightly larger than that of males. The beak is straight and long. Unlike most other ziphiids, they have two pairs of teeth in the lower jaw. The blowhole slit is unique, with a posteriorly opened arch that is unlike those of all other odontocete groups (e.g. Kasuya^18^). Although the nasals are large, they do not overhang the superior nares.

## History of ***Berardius*** in Japan

In 1910, True^11^ summarised the ziphiid specimens that were preserved and stated “*Berardius* is the rarest genus, only about fourteen specimens having been collected thus far”. Also in 1910, Andrews visited the Imperial Museum at Tokyo, which is now called the National Museum of Nature and Science (NMNS), to find a *B*. *bairdii* skeleton^19^; this occurred when existence of *Berardius* in Japan was known to science and, on this historical occasion, *B*. *bairdii* was confirmed to correspond to “tsuchi-kujira”^20^ of Japan. When considering the recognition of *B*. *bairdii* in Japan, however, the Japanese name tsuchi-kujira had been used since the early 18th century, and whaling activities have been aimed at this species since then^21–24^. Proper comparison and recognition of this species using the Western (or Linnean) systematic scheme took some time after the introduction of modern science from the West, which began in 1868 after the Meiji Restoration. Researchers such as Okada^25^ incorrectly identified tsuchi-kujira as *Hyperoodon rostratus*, and this notion was generally accepted in most publications. In 1910, Andrews examined the specimens of tsuchi-kujira (then recognised as *H*. *rostratus*) that were exhibited in the Imperial Museum in Tokyo, and identified them as *B*. *bairdii*^19^. He surveyed the locality of this *B*. *bairdii* specimen and collected a whole skeleton of this species in Chiba. This event was reported by Nagasawa^20^ to the Zoological Society of Japan and confirmed the existence of *B*. *bairdii* in Japanese waters.

## Results

The following description was prepared by Tadasu K. Yamada, Shino Kitamura and Takashi F. Matsuishi.

### Systematics

Order CETARTIODACTYLA Montgelard, Catzeflis and Douzery, 1997^26^.

Infraorder CETACEA Brisson, 1762^27^

Parvorder ODONTOCETI Flower, 1864^28^

Family ZIPHIIDAE Gray, 1865^29^

*Genus BERARDIUS* Duvernoy, 1851^8^

*Berardius minimus* sp. nov.

(New Japanese name: Kurotsuchikujira)

### Etymology

**Figure 1:**
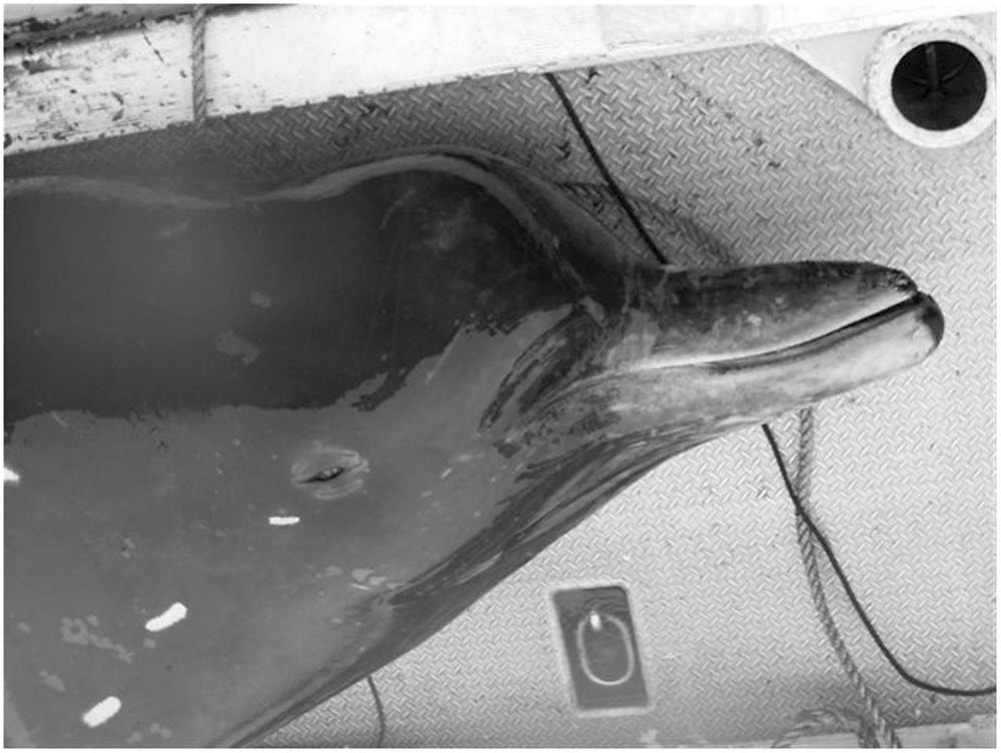
Unidentified beaked whale incidentally caught in Shibetsu, Hokkaido (photo taken by Minako Kurasawa on 20 July 2004, courtesy of Hal Sato).

**Figure 2:**
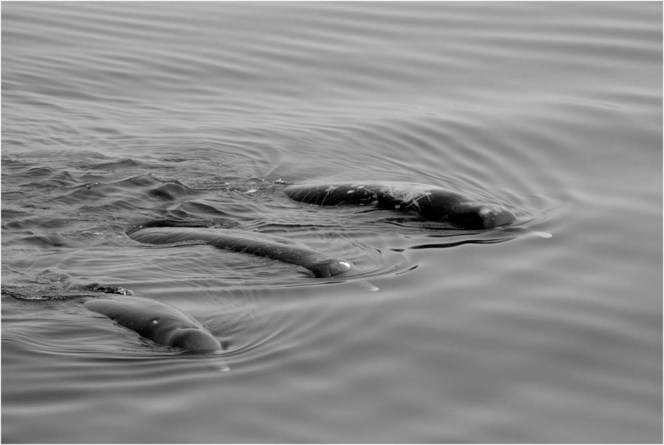
Unidentified beaked whales sighted in Nemuro strait. Note the short beak, dark body colour, and sparse linear scars (photo taken by Hal Sato on 21 May 2009).

The specific name reflects the smallest body size of physically mature individuals of this species compared with the other *Berardius* species. Historically, whalers in Hokkaido recognised this species as different from *B*. *bairdii* and called them “kuro-tsuchi”, which means black Baird’s beaked whale; however, the colour difference mainly depends on the scar density and is not biologically fundamental (Figs 1 and 2). We therefore chose the most basic difference, the significantly small body size, which is smallest among the congeners, to be reflected in the scientific name.

### Holotype

**Figure 3:**
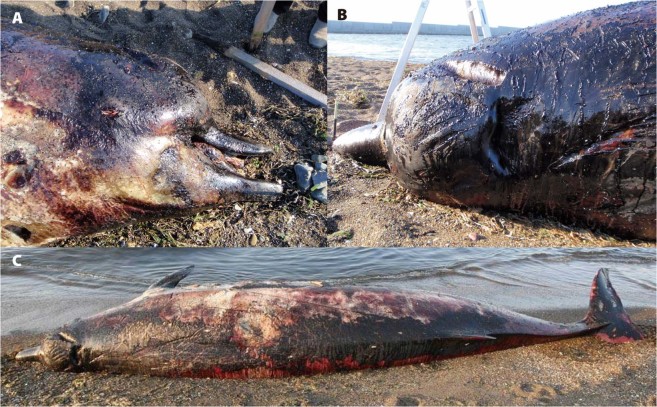
Severely decomposed beaked whale stranded in Kitami, Hokkaido on 4 June 2008. (**A**) The relatively shorter beak indicates it is not *B*. *bairdii* (photo taken by Mari Kobayashi), (**B**) although the blow hole shape indicates it belongs to *Berardius* (photo taken by Mari Kobayashi). (**C**) The general body shape is that of typical ziphiid species. When compared with adult *B*. *bairdii*, this specimen is more spindle-shaped.

Adult male (NSMT-M35131) skull, mandible, and most of post of postcranial skeleton at National Museum of Nature and Science (NMNS). In addition, tissue samples are also preserved at the NMNS. This specimen, a fairly well decomposed stranded carcass was found on 4 June 2008 (Fig. 3A–C). Upon receiving notice, SNH took action, and Prof. Mari Kobayashi of Tokyo University of Agriculture and her students examined the carcass on-site. The carcass was then buried at a nearby. The whole skeleton was excavated and recovered on 26 and 27 August 2009 by one of us (SNH), Tokyo University of Agriculture, Institute of Cetacean Research, and NMNS.

### Type Locality

Tokoro Town (44°07′14.5N, 144°06′29.6E), Kitami City, Hokkaido, Japan, southern Okhotsk Sea, North Pacific.

### Nomenclatural statement

A Life Science Identifier (LSID) was obtained for the new species (*B. minimus*): urn:lsid:zoobank.org:act:C8D63A76-B1A3-4C67-8440-AFCE08BE32E9, and for this publication: urn:lsid:zoobank.org:pub:52AD3A26-4AE6-42BA-B001-B161B73E5322.

### Diagnosis

*Berardius minimus* differs from all of its congeners by having the following unique characters: remarkably smaller body size of physically mature individuals, proportionately shorter beak, darker body colour subsequent noticeable cookie-cutter shark bites.

### External characters

**Figure 4:**
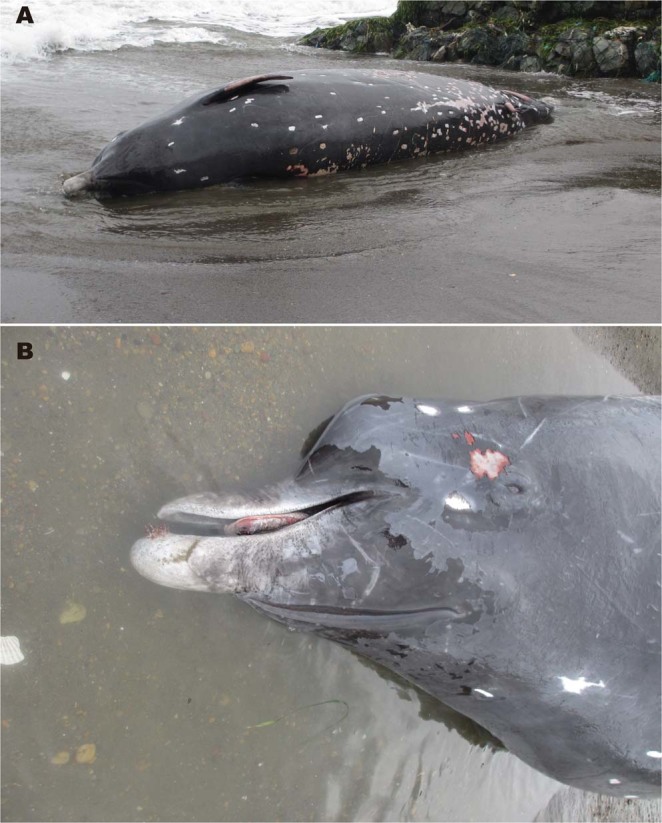
Fresh carcass of *Berardius minimus* (male, 662 cm) found stranded on 10 November 2012 in Sarufutsu Hokkaido. (**A**) Ventral view of the carcass. Note the whole body is almost black except for the faintly white beak. (**B**) The relatively short beak of the same individual (photos taken by Yasushi Shimizu).

External appearance is mostly known from a male individual found stranded on 10 November 2012 in Sarufutsu, Hokkaido (Fig. 4). Most of the external characters of *B*. *minimus* are typical of medium- to large-sized ziphiids, with several discriminating characters, such as the narrow, straight, and longer beak; reverse V-shaped throat grooves; relatively smaller flippers (flipper length is 11.4% of body length on average; range, 7.7–13.4%); small dorsal fin (dorsal fin height is 3.7% of body length on average; range, 3.4–3.9%) located 70% of body length (on average; range, 66.7–71.8%); and tail flukes that lack the median notch. However, the posteriorly opened crescent-shaped blowhole slit indicates *Berardius* affinity. Additionally, *B*. *minimus* has a substantially smaller body size (maximum body length of 6.9 m in physically mature individuals, so far), more spindle-shaped body, and relatively shorter beak, which is approximately 4% of the body length and is not consistent with the morphology of either of the known *Berardius* species.

Body colour is almost black with a pale white portion on the rostrum; this is in contrast to *B*. *bairdii*, which is described as “slatish”^4^ or “slate grey”^6,7^ or *B*. *arnuxii*, which is described as black^30^ or light grey^31^. The greyish tone of the *B*. *bairdii* body is mainly attributed to the dense healed scars that are probably caused by intraspecific conflicts and/or behaviour. At least in adult and subadult individuals of *B*. *minimus*, cookie-cutter shark bites are fairly conspicuous, but not to the extent as usually seen in some other species such as *Ziphius cavirostris*, *Mesoplodon densirostris*, and/or *Balaenoptera borealis*. The darker body colour with almost no scars produces a sharp contrast with the healed cookie-cutter shark bites, which are white and very conspicuous against the black body of *B*. *minimus*.

**Figure 5:**
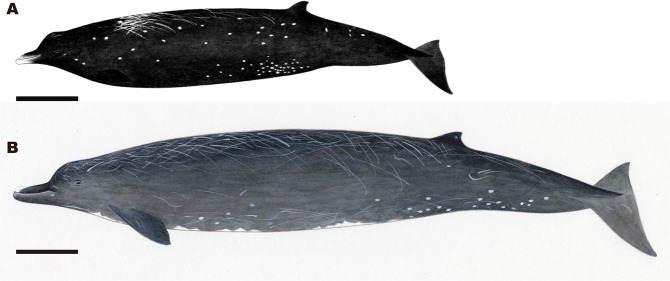
Illustrations of (**A**) *Berardius minimus*, and (**B**) *B*. *bairdii*. The black bars show 1 m. In general appearance, *B*. *minimus* resembles a small *B*. *bairdii* with a proportionately shorter beak and more spindle-shaped body (drawn by Yoshimi Watanabe, National Museum of Nature and Science).

The beak is much shorter than in the other two *Berardius* species. In *B*. *bairdii*, the head proportions are extremely small, and are much smaller than that of *B*. *minimus*. Body colour is almost uniformly dark brown with a whiter portion at the tip of rostrum. No white patch on the belly was confirmed in *B*. *minimus*. An illustration of an adult male of *B*. *minimus* is shown as Fig. 5. At present, we do not know what adult females look like.

### External measurements

As mentioned above, the distinctly small body length of physically mature individuals and proportionately shorter beak are the most reliable characters which indicate that the population in question represents a species that was previously not known to science.

Regarding body length, a strong significant difference was found between the body length of male *B*. *bairdii* from the Okhotsk Sea (n = 34)^32^ and mature male *B*. *minimus* (n = 4, Table 1) (Welch’s t-test, t = 18.5, P < 0.001).

**Table 1:** List of *Berardius minimus* specimens that were stranded or drifting and collected in Hokkaido.

No.	Specimen ID		SNH ID	Sex	Body Length cm	Found date		Locality		Latitude Longitude	stranding	Specimen	Growth Stage	Analyses
1	NSMT	M35131	08019	M	660	2008.06.04	Japan	Hokkaido	Kitami	44°07′14.50N 144°06′29.60E	Stranding	complete skeleton	Physically mature	*,†,‡
2	NSMT	M36219	09009	F	U	2009.05.11	Japan	Hokkaido	Rausu	44°00′49.80N 145°14′72.00E	Drifing	severed head	Neonate?	‡
3	NSMT	M35206	09016	F	621	2009.06.17	Japan	Hokkaido	Utoro	44°02′18.40N 144°56′01.30E	Stranding	complete skeleton	Physically immature	†,‡
4	NSMT	M42000	12044	M	630	2012.08.23	Japan	Hokkaido	Rausu	44°09′22.07N 145°17′33.03E	Drifting	almost complete skeleton	Physically mature	*,†,‡
5	NSMT	M42012	12054	M	662	2012.11.10	Japan	Hokkaido	Sarufutsu	45°20′21.30N 142°10′09.27E	Stranding	complete skeleton	Physically mature	*,†,‡
6	NSMT	M42610	14016	M	690	2014.06.14	Japan	Hokkaido	Rausu	44°05′56.94N 145°18′38.16E	Drifting	complete skeleton	Physically mature	*,‡

*Indicates individuals used for body length analysis, ^†^for external measurement comparison, and ^‡^for molecular phylogenetic analysis.

To confirm relative rostrum-to-body length, Welch’s *t*-test was also conducted. For *B*. *minimus*, four samples in Table 1 were analysed. For *B*. *bairdii*, the mean and standard deviations for male *B*. *bairdii* in the Okhotsk Sea (n = 29) that appeared in Table 2 of Kishiro^32^ were used. Rostrum length was standardised by body length, and was 3.62 ± 0.39 SD% (n = 4) for *B*. *minimus* and 5.81 ± 0.80 SD% (n = 29) for *B*. *bairdii*. Welch’s *t*-test showed strong significant difference (P = 2.3 × 10^−5^). Female *B*. *bairdii* relative length was 6.27, which is longer than that of males. Note this female was not physically mature. The difference between *B*. *minimus* and *B*. *bairdii* was obviously larger if the sex-pooled data were used. A strong significant difference was also found between *B*. *minimus* and *B*. *bairdii* in the Pacific Ocean and Sea of Japan (P < 0.001). Thus, the relative rostrum length of *B*. *minimus* was significantly shorter than that of *B*. *bairdii*. However, we note that the sample size for both *B*. *minimus* and *B*. *arnuxii* are extremely small, in contrast to *B*. *bairdii*.

**Table 2:** Mean, standard deviation, and range of each measurement by species.

			*Berardius minimus* n = 4				*Berardius bairdii* n = 10				*Berardius arnuxii* n = 7			
			mean	s.d.	min	max	mean	s.d.	min	max	mean	s.d.	min	max
Measurement items	SI1		970	42.9	935	1,042	1,346	76.5	1,158	1,403	1,314	100.3	1,161	1,410
	SI2		888	36.1	861	949	1,211	63.8	1,089	1,287	1,148	91.3	1,023	1,253
	SI3	Left	656	47.5	617	737	953	59.7	805	1,006	925	73.4	794	1,023
	SI4	Left	754	44.7	716	827	1,085	47.7	973	1,148	1,038	86.6	891	1,137
	SI5		770	39.7	732	835	1,097	61.8	967	1,183	1,076	96.3	915	1,190
	SI6	Left	915	30.9	890	968	1,215	78.1	1,027	1,298	1,194	98.8	1,053	1,301
	SI7		609	37.2	577	670	864	54.6	725	921	835	71.9	696	918
	SI11		280	10.4	268	292	377	33.4	314	434	362	26.5	325	394
	SI12		411	10.7	394	422	521	33.2	456	573	522	46.2	442	572
	SI13		173	13.3	151	186	227	14.6	207	258	211	23.2	179	248
	SI16		60	7.4	49	69	78	8.8	60	86	76	3.5	72	82
	SI18		101	9.4	86	111	118	18.2	103	163	121	11.8	106	140
	SI20		91	6.6	84	102	112	7	103	125	132	11.2	118	155
	SI23		75	2.9	71	79	96	9.1	74	111	93	7.4	84	108
	SI25		132	31	79	157	225	9.9	210	241	214	17.1	195	249
	SI28		135	14.3	118	157	155	9	134	169	166	14.1	141	183
	SI29		61	4.3	55	67	80	5.7	70	88	90	8.6	78	104
	SI31		125	8	112	132	194	14	163	216	176	14.5	160	199
	SI32		65	1.5	63	67	112	8.9	94	126	99	10	86	115
	SI33		64	3.7	58	67	97	5.9	89	105	90	10.3	77	104
	SI36	Left	145	8.5	133	154	174	19.6	151	208	219	22.3	179	258
	SI37	Left	84	14	68	106	108	13.1	83	123	110	13.9	86	128
	SI39		548	46.8	497	624	794	54	665	869	794	74.9	682	908
	SI40	Left	521	31.3	485	565	858	226.2	647	1,305	750	62.2	656	818
	SI41		491	30.3	461	534	737	48.4	623	787	717	63.4	618	781
	SI42		723	44.8	680	796	1,018	58.3	858	1,078	995	78.9	858	1,070
	SI45		115	2.9	110	117	166	12.6	152	197	163	8	152	175

### Skull morphology

**Figure 6:**
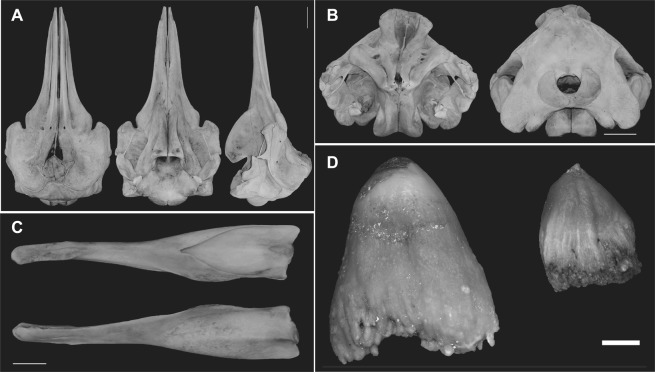
Skull of the *B*. *minimus* holotype. (**A**) Dorsal, lateral, and ventral views of the skull. Note the relatively short rostrum. The white bar indicates 10 cm. (**B**) Anterior and posterior views of the skull. The dorsal view is more triangular, whereas the dorsal views in *B*. *bairdii* and *B*. *arnuxii* are more pentagonal. The white bar indicates 10 cm. (**C**) Lingual (inner, upper) and buccal (outer, lower) sides of the left mandible. The white bar indicates 10 cm. (**D**) Buccal (external) view of the anterior (left) and posterior (right) teeth of the lower jaw. The white bar indicates 1 cm.

The skull morphology resembles the skulls of both existing *Berardius* species, but *B*. *minimus* has a distinctly shorter rostrum if contrasted to the condylobasal length, and smaller bulla and periotic bone. In general, the sutures are more tightly closed in *B*. *minimus* than those in the other *Berardius* species. In the hyoid bone, thylohyal and basihyal are not fused at all (Fig. 6).

#### Superior aspect

The following characters are readily recognisable as species-specific. The relative beak length in *B*. *minimus* is clearly smallest among the three *Berardius* species. The *B*. *minimus* skull has much tighter sutures compared with those in both *B*. *arnuxii* and *B*. *bairdii*. The proportional distance of the anterior end of the maxillae from the tip of the rostrum (i.e. premaxillae) relative to condylobasal length of the skull is much smaller in *B*. *minimus* (6.93% in NSMT35131) than the two previously known *Berardius* species (which have a distance of approximately 10%). The inclination of the occipital bone is stronger in *B*. *minimus*, and the occipital plane is much wider compared with the other two species. The antorbital notch is proportionately narrower in *B*. *minimus* than in *B*. *bairdii* but similar to that in *B*. *arnuxii*. The *B*. *minimus* rostrum has simple tapering contour lines toward the tip, whereas both contour lines of the rostrum are parallel in *B*. *bairdii* and *B*. *arnuxii*. The lateral border of the orbit, which consists of the maxilla and frontal bones, is almost parallel to the sagittal plane in *B*. *minimus*, but is oblique in other two species.

#### Lateral aspect

The relative rostrum length is obviously shorter in *B*. *minimus*, and the *B*. *minimus* rostrum also looks much shorter than those of the other two species in side view. The skull height relative to condylobasal length is much larger (0.41–0.44) in *B*. *minimus* than those in *B*. *bairdii* (0.35–0.40) and *B*. *arnuxii* (0.40–0.41). There is stronger inclination of the higher portion of the occipital plane in *B*. *minimus*, and the convexity of the occipital plane is stronger in *B*. *minimus*. The temporal fossa is the shallowest in *B*. *minimus* and the medial wall of the fossa is convex, but is concave in *B*. *bairdii* and *B*. *arnuxii*.

#### Posterior aspect

The structure above the temporal fossa is proportionately much larger and higher in *B*. *minimus* than those in *B*. *bairdii* and *B*. *arnuxii*, which gives the impression that the *B*. *minimus* skull is rather triangular in the posterior view, whereas those of the other two species are pentagonal.

#### Anterior aspect

In the frontal view, lateral expansion of the premaxillae at the posterior is prominent, and the posterior margins of both maxillae are clearly visible in *B*. *minimus*.

In *B*. *minimus*, the height of skull relative to the width is much higher than those of the other *Berardius* species. The prominential notch and related structure are much higher, more distinct and more rugged in *B*. *bairdii* and *B*. *arnuxii*.

#### Teeth

As in the other two *Berardius* species, *B*. *minimus* has two pairs of teeth only at the tip of the lower jaw. The anterior tooth is much larger than the posterior tooth. Teeth dimensions of the holotype are shown in Table 3 (57-1 and 2, 58-1 and 2). In the holotype specimen of *B*. *minimus* the pulp cavities are almost closed in all teeth other than the right 2^nd^ tooth, where the pulp cavity is open.

**Table 3:**
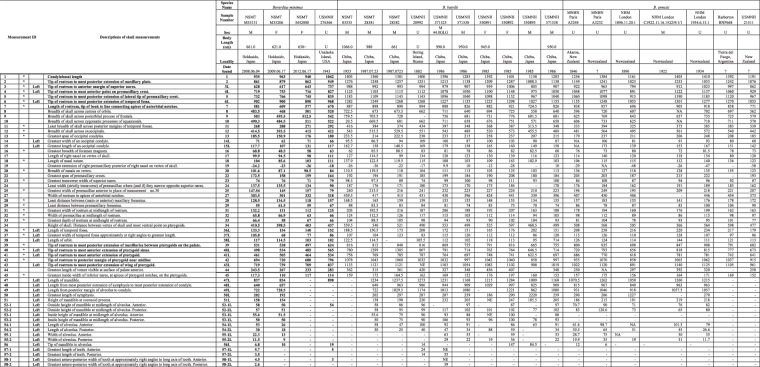
Skulls used for craniometry analyses. Twenty-one specimens (10 *Berardius bairdii*, seven *B*. *arnuxii*, and four *B*. *minimus*).

Specimens are stored at National Museum of Nature and Science (NMNS), United States National Museum of Natural History (USNMH), Natural history Museum of London (BMNH), le Museum National d’Histoire Naturelle (MNHN), and Museo Acatushún (MA). Items with * were used for multivariate analysis.

#### Post cranial skeleton

**Figure 7:**
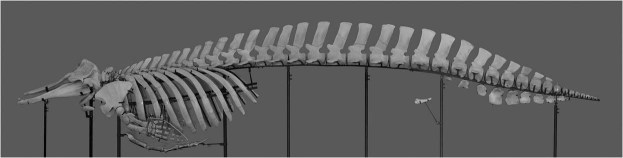
Articulated skeleton of the *B*. *minimus* holotype specimen.

The vertebral column has proportionately high spinous processes, which is observed in most ziphiid species (Fig. 7). The bone matrix is coarse and porous, and they float on the processing water after internal soft tissue was removed. In the holotype specimen, the vertebral formula is C. 7, Th. 10, L. 10, Ca. 19, making the total count as 46. Among 7 cervical vertebrae, C1–C3 were fused. L4 and L5 are the tallest vertebrae. Ca10 and 11 are so-called ball vertebrae. Ten chevrons were counted. Ribs are in 10 pairs, among which seven pairs are dual-headed with both costovertebral and costotransversal articulations. The remaining three pairs have only one articulation facet which articulate with “transverse” processes of the caudal thoracic vertebrae. No ossified cervical ribs were found. The sternum is composed of five segments.

Paired ossified pelvic bones have a lateral surface which is fairly smooth; however, in the medial surface, approximately two-thirds of the total length is an elevated area where the corpus cavernosum penis attaches. Viewed from the dorsal side, the pelvic bones show a very gentle s-shape. No rudimental femur or any additional appendicular bone was collected.

#### Pectoral appendage

Regrettably, we could not secure all phalangeal bones of the left flipper. On the right side, there are three carpal bones in the proximal row, possibly the Ossa radiale, centrale, and ulnare. In the distal row are another three carpal bones. All five digits have one each metacarpal; the phalangeal formula is 0-5-4-3-2.

### Multi-measurement comparison

Table 2 shows the mean, standard deviation, and range of each measurement by species. PCA showed that the contribution of the first principal component (PC1) was 73.9%, and the cumulative contribution reached 90% for PC1-6. Thus, linear discriminant analysis was conducted using PC1-6.

**Figure 8:**
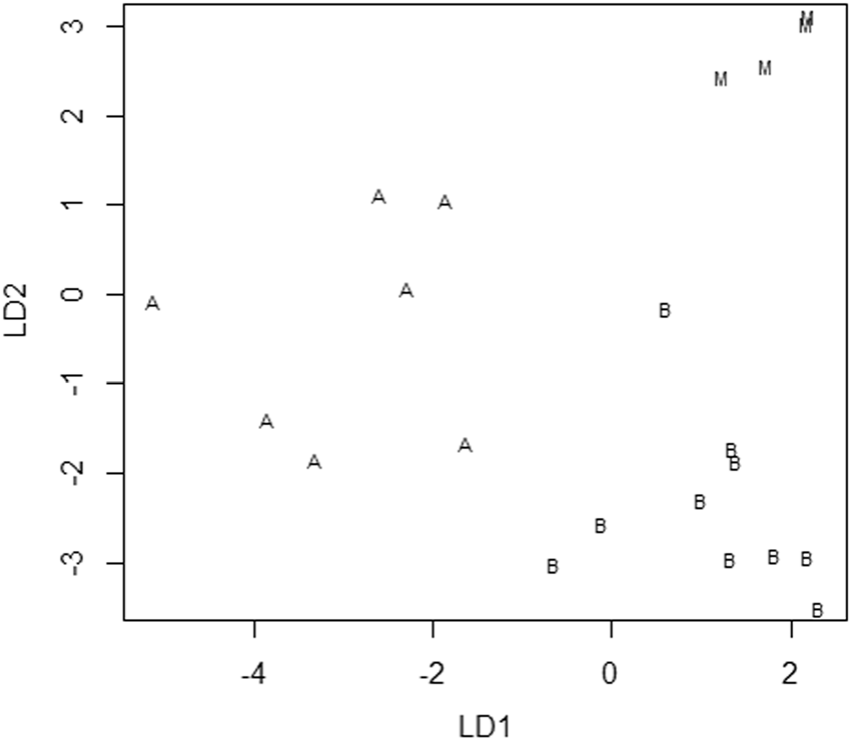
Linear discriminant variates of each sample are plotted. The linear discriminants variates are clearly separated by species. B: *Berardius bairdii*, A: *B*. *arnuxii*, M: *B*. *minimus*.

Table 4 shows the linear discriminant coefficients obtained by linear discriminant analysis (LDA). The linear discriminants coefficients of each sample are plotted in Fig. 8. The distribution of the linear discriminants variates was very clearly separated by species.

**Table 4:** Linear discriminant coefficients obtained by linear discriminant analysis (LDA).

	LD1	LD2
PCA1	−0.101	0.223
PCA2	−0.774	−0.348
PCA3	0.416	0.065
PCA4	−0.101	0.223
PCA5	−0.774	−0.348
PCA6	0.416	0.065

### Genetic considerations

**Figure 9:**
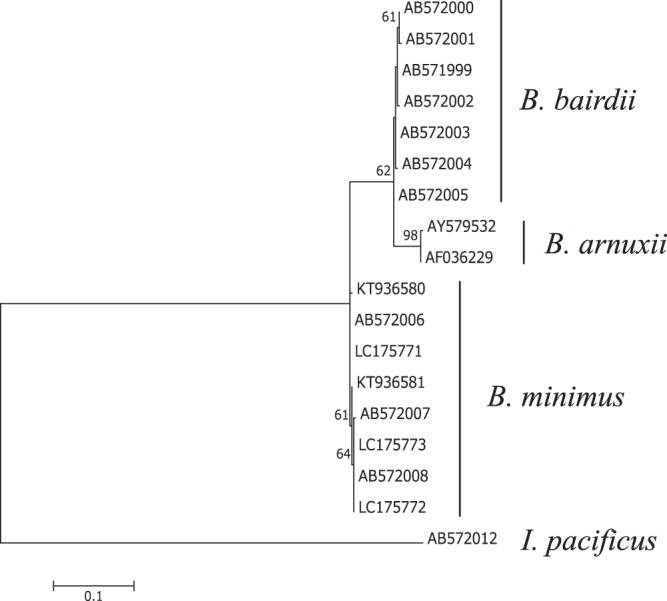
Maximum likelihood-based molecular phylogenetic relationships among the three *Berardius* species, with *Indopacetus pacificus* as the outgroup. See Materials and Methods for details regarding nucleotide sequencing and tree construction.

Molecular phylogenetic relationships among three *Berardius* species were examined using nucleotide sequence variation of the mitochondrial (mt)DNA control region (CR). The 879-bp complete CR sequence data from eight *B*. *minimus* specimens (Table 5) (Acc. Nos AB572006-AB572008 from Kitamura *et al*.^6^, Acc. Nos LC175771-LC175773 in this study, and Acc. Nos KT936580-KT936581 from Morin *et al*.^7^) showed five haplotypes with only 1–4 nucleotide differences without gaps after multiple alignment. Using the CR sequences aligned with 430-bp *B*. *arnuxii* sequences (Acc. Nos AF036229 and AY579532 from Dalebout *et al*.^16^) excluding gaps, the number of nucleotide differences between *B*. *minimus* and its congeners was 18–22 for *B*. *bairdii* and 25–29 for *B*. *arnuxii*. Thus, the mtDNA nucleotide difference between *B*. *minimus* and any of its congeners was much greater than the difference between *B*. *bairdii* and *B*. *arnuxii*, which is 12–16 nucleotides. The observed CR nucleotide differences supported the distinct position of *B*. *minimus* in the *Berardius* tree constructed from 430-bp sequences using the maximum likelihood method, where *B*. *bairdii* and *B*. *arnuxii* formed a sister clade (Fig. 9).

**Table 5:** Individuals and sequences used in this study. SNH: Stranding Network Hokkaido, Hokkaido, Japan; EW: Ehime University es-Bank, Ehime, Japan; NSMT: National Museum of Nature and Science, Ibaraki, Japan.

Code	Haplotype No.	Acc. No.	Reference
***B***. ***minimus***			
SNH08019	1	AB572006	Kitamura *et al*.^6^
SNH09009	2	AB572007	Kitamura *et al*.^6^
SNH09016	3	AB572008	Kitamura *et al*.^6^
SNH12044	1	LC175771	this study
SNH12054	3	LC175772	this study
SNH14016	3	LC175773	this study
	4	KT936580	Morin *et al*.^7^
	5	KT936581	Morin *et al*.^7^
***B***. ***bairdii***			
EW01000	1	AB571999	Kitamura *et al*.^6^
EW01005	2	AB572000	Kitamura *et al*.^6^
EW00997	3	AB572001	Kitamura *et al*.^6^
EW01015	4	AB572002	Kitamura *et al*.^6^
EW01007	5	AB572003	Kitamura *et al*.^6^
EW00999	6	AB572004	Kitamura *et al*.^6^
EW01004	7	AB572005	Kitamura *et al*.^6^
***B***. ***arnuxii***			
	1	AF036229	Dalebout *et al*.^16^
	2	AY579532	Dalebout *et al*.^16^
***I***. ***pacificus*** (**outgroup**)			
NSMT M33006	1	AB572012	Kitamura *et al*.^6^

### Known distribution

**Figure 10:**
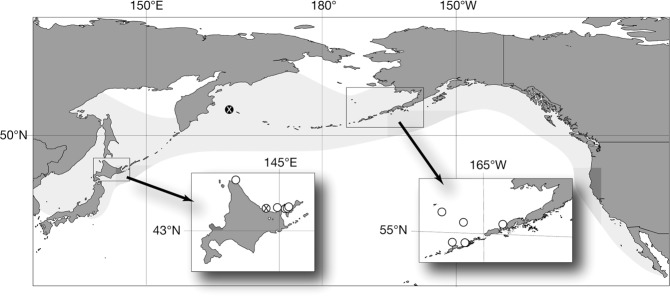
*Berardius minimus* localities plotted against the *B*. *bairdii* distribution map (shaded area, as described by Kasuya^18^). Circles show *B*. *minimus* localities. The white circle with a black X indicates the *B*. *minimus* type locality, whereas the black circle with the white X indicates the *B*. *bairdii* type locality.

As is indicated by the map of localities where *B*. *minimus* was found (Fig. 10), their known distribution is very limited and occurs between 40°N and 60°N, and 140°E and 160°W.

## Discussion

Kasuya^5,18^ summarised Hokkaido whalers’ traditional knowledge. The whalers recognised two types of tsuchi-kujira: the ordinary “tsuchi-kujira” (*Berardius bairdii*) and the darker and smaller “kuro-tsuchi” (black Baird’s beaked whale) or “karasu” (crow). However, it is unclear whether “kuro-tsuchi” and “karasu” are used to describe the same type of whales or each notion represents the different population.

In this study, we described a new species, *B*. *minimus*, which corresponds to “kuro-tsuchi”. If “karasu” exists as a third type, it could be a species that is not yet recognised or a *Mesoplodon* species found in Hokkaido (either *M*. *stejnegeri* or *M*. *carlhubbsi*). Recognition of these *Mesoplodon* species around Hokkaido is rather recent; the earliest *M*. *stejnegeri* specimen was collected in 1985^33^, and the earliest *M*. *carlhubbsi* in 2004^34^. These *Mesoplodon* species were not recognised as distinct species by whalers or the media until recently.

As was also pointed out by Kasuya^18^, Fig. 364 and 366 of Heptner^35^ hinted at the possibility of a *Hyperoodon*-like whale in the northern Pacific. The animal in the photo was definitely not *Berardius*. This could be a species of probably about 10-m long with a beak almost like that of *Hyperoodon*. We suspect this could be an example of an extralimital occurrence of *H*. *ampullatus*. Considering the recent sightings of the gray whales in the Mediterranean or in Namibia^36,37^, the possibility of vagrant individual navigate through the Northwest passage during summer should be studied.

The species we described is rather readily recognisable by people with whale taxonomy experience based on the external characters. The species has an obviously smaller body size, which is 6.3–6.9 m in physically mature individuals, so far we confirmed (Morin *et al*.^7^ reported an adult male with 7.3 m body size). Their body size ranges from 9.1–11.1 m in *B*. *bairdii* and 8.5–9.75 m in *B*. *arnuxii*^38^. They have a relatively short beak that is approximately 4% of the body length. They have a dark body colour, which is almost uniformly black with noticeable healed cookie-cutter shark bites forming white dots; this impressively contrasts with the much lighter colouration of *B*. *bairdii* and likely result from healed scratches and scars that were probably caused by intra-specific struggling and bottom-feeding behaviour.

Osteologically, the small body size of physically mature individuals is the main defining character of *B*. *minimus*. Condylobasal length of the skull is 935–1042 mm, in contrast to 1343–1524 mm in *B*. *bairdii* and 1174–1420 mm in *B*. *arnuxii*^9^. Skull characters indicate significant influence of size difference, such as tighter bone sutures compared with those of other *Berardius* species. Skull elements of the brain case are relatively large and conspicuous. The vertebral formula of the type specimen is C. 7, Th. 10, L. 10, Ca. 19 (totalling 46), whereas it is C. 7, Th. 9–11, L. 12–14, Ca. 17–22 (47–52) in *B*. *bairdii* and C. 7, Th. 10–11, L. 12–13, Ca. 17–19 (47–49) in *B*. *arnuxii*^13^. Rib count, which reflects the thoracic vertebral count, is 10 in the *B*. *minimus* type specimen.

As was mentioned above, when comparing the skull sutures in similarly mature individuals of different species of cetaceans, there is a general tendency that the larger the adult form is the less rigid skull composition is observed. Cetacean facial skull consists loosely articulated bones, including the maxillae, premaxillae and frontals, which are adhered to the mesorostral cartilage pillar on the vomer by connective tissue. It is a physically significant principle where cetaceans swing their rostrum in the water for foraging. It requires tremendous power and the flexibility of the skull structure must ease the stress given to the skull structure. In this context it is quite reasonable that the skull of *B*. *minimus* is far more rigidly composed compared to those of the far larger species, such as *B*. *bairdii* and *B*. *arnuxii*. It means adult size of *B*. *minimus* is essentially far smaller than the other two *Berardius* species.

The molecular biology of *B*. *minimus* was previously discussed by Kitamura *et al*.^6^, and specific genome characters were only identified in individuals collected from Hokkaido. However, we found a skull with *B*. *minimus* characters in the collection of the USNM which was collected from the Unalaska Island in 1943. Additional individuals were detected among the samples collected in the Aleutian area, and further analyses and considerations were conducted and discussed by Morin *et al*.^7^. Further detailed analyses on *Berardius* species in both the northern and southern hemispheres are needed to explain *Berardius* speciation processes.

The currently recorded *B*. *minimus* distribution is very limited and occurs between 40°N and 60°N, and 140°E and 160°W. They have fairly dense cookie-cutter shark (*Isistius brasiliensis*) bites. The cookie-cutter shark is understood to be a tropical to warm-temperate species and their northern limit in the western North Pacific is reported to be 30°N to 43°N^39^. However, the southern limit of the *B*. *minimus* distribution might extend further south.

Although species identities of *B*. *arnuxii* and *B*. *bairdii* have been previously debated, we described another species of this genus. However, it is unclear whether *B*. *minimus* speciation occurred before or after the antitropical split of *B*. *arnuxii* and *B*. *bairdii*. Additionally, the area where *Berardius* speciation took place should be examined in the future.

## Methods

### Specimens examined

The specimens of this unknown species, which were collected in Hokkaido, are listed in Table 1. No live animals were used for the current research. Observations on the external appearance and morphometrics, observations on the skeleton especially of the skull, skull morphology and measurements and molecular phylogenetic analysis were conducted.

### External morphology and measurements

External observations of the five individuals of *Berardius minimus* (three physically mature males, one subadult female, and a head of one neonate female) were made, and the external morphometrics following previous studies^32,40^ (Tables 6 and 7) were conducted on four *B*. *minimus* (all physically mature males; Table 1). Raw data examination revealed that body length and the ratio of beak length-to-body length significantly differed, and Welch’s t-test was applied to these variables.

**Table 6:** External measurements of *Berardius minimus* used for the comparison with *B*. *bairdii*.

	Specimen ID	M35131	M35206	M42012	M42610
	Sex	M	F	M	M
V1	Body length from tip of snout to notch of flukes	660	621	662	690
V2	Tip of snout to tip of dorsal fin	473	429	475	467.1
V3	Tip of snout to blow hole	65	68	77	53
V4	Length of snout	25	25.2	22.9	21.8
V5	Projection of lower jaw beyond tip of snout	—	5	6	5.1
V6	Tip of snout to angle of gape	36	35	44	38
V7	Tip of snout to centre of eye	59	63.5	60	53
V8	Tip of snout to anterior insertion of flipper	115	105	105	110
V9	Tip of snout to umbilicus	—	273	307	317.3
V10	Tip of snout to centre of genital aperture	432	425	461.5	464.3
V11	Tip of snout to anus	483	449	497	477
V12	Centre of eye to centre of ear	—	13	24.7	16.6
V13	Fluke length from anterior insertion to notch	55	56.5	60	54.6
V14	Fluke width from tip to tip	167	162	179	176.2
V15	Length of base of dorsal fin	70	65	56.5	48
V16	Vertical height of dorsal fin	25	22	22.5	27
V17	Maximum width of flipper	28	23.5	25.3	25.9
V18	Straight length of flipper from tip to anterior insertion	77	48	85.2	92.8

**Table 7:** Measured external morphometrics characters for *B*. *bairdii* as described in previous studies^32,40^.

Measurements		n	Mean (cm)	SD
Measurement items listed in Table 6	V1	34	997.8	78.82
	V2	22	703.5	68.89
	V3	28	107.4	11.1
	V4	29	58	8.02
	V5	23	7.2	2.72
	V6	27	62.2	6.78
	V7	23	93.5	11.04
	V8	24	160.4	20.19
	V9	24	438	33.5
	V10	23	641.8	57.23
	V11	24	711.4	61.06
	V12	22	21.7	1.79
	V13	20	81.5	10.3
	V14	10	271.9	15.44
	V15	19	58.2	9.35
	V16	20	25.1	2.92
	V17	19	40.8	3.55
	V18	16	123.6	7.56

### Skeletal morphology and measurements of the skull

Observations of the skeleton, especially of the skull, and skull measurements were made for 21 specimens (10 *B*. *bairdii*, seven *B*. *arnuxii*, and four *B*. *minimus*) (Table 2). Specimens are stored at the USNM, NMNS, MNHN, Natural History Museum of London (BMNH), and Museo Acatushún (MA).

### Multivariate analysis

To examine the difference between the morphological features among species, a multivariate analysis was conducted. To describe the effect of the difference of body size by species, a principal component analysis (PCA) was conducted for 27 measurements shown in Table 5 for 22 samples (four *B*. *minimus*, 10 *B*. *bairdii*, seven *B*. *arnuxii*) shown in Table 3. For all variables, measured values using this analysis are indicated in bold gothic.

A linear discriminant analysis (LDA) was then conducted to compare species using the scores obtained from the principal component analysis (PCA). Calculations were carried out using “prcomp” and “lda” function in R ver.3.3.1^41^.

### Nucleotide sequence analysis and molecular phylogeny

The 18 mtDNA control region (CR) sequences analysed (Table 5) included sequences from three *B*. *minimus* specimens (Acc. Nos LC175771-LC175773 for SNH12044, SNH12054, and SNH14016, respectively) and 15 previously reported sequences, which included seven for *B*. *bairdii* (Kitamura *et al*.^6^, AB571999-AB572005), five *B*. *minimus* (Kitamura *et al*.^6^, AB572006-AB572008, updated complete sequences August 2016; and Morin *et al*.^7^, Acc. Nos KT936580-KT936581), two *B*. *arnuxii* (Dalebout *et al*.^16^, Acc. Nos AF036229 and AY579532), and one *Indopacetus pacificus* (Kitamura *et al*.^6^, AB572012) as an outgroup. *I*. *pacificus* was selected because it belongs to the same family but is in a rather distant genus, which was inferred by a previous CR phylogenetic tree^6^. All the newly collected samples for the nucleotide sequence analysis and molecular phylogeny were officially transferred to the authors from the original sample holder, the Stranding Network Hokkaido. Nucleotide sequencing of the complete mtDNA CR in the three *B*. *minimus* was performed using primer pairs CRL (5′-CAA CAC CCA AAG CTG GAA TTC T-3′)^6^ and CRH2 (5′-TAG ACA TTT TCA GTG TCT TGC-3′, which was newly designed for this study) for PCR amplification, and CRH (5′-CCA TCG AGA TGT CTT ATT TAA G-3′)^6^ and LCR (5′-GAC ATC TGG TTC TTA CTT CAG G-3′)^42^ as internal sequencing primers.

CR sequence alignment was performed using CLUSTAL X^43^, and the output was inspected by eye following the application of multiple alignment parameters in the program. All CR sequences were adjusted to the short length of the *B*. *arnuxii* sequence, 430 bp (Dalebout *et al*.^16^, Acc. Nos AF036229 and AY579532), for multiple sequence comparison and molecular phylogenetic analysis.

A molecular phylogenetic tree was constructed with 430-bp mitochondrial CR sequences of all analysed species using the maximum likelihood algorithm in MEGA version 7^44^ based on the Tamura 3-parameter model^45^ with gamma distribution (parameter = 0.2001), which was suggested to be the best nucleotide substitution model based on a model test in this program. Bootstrap values were calculated by 1,000 replicates^46^.

## Data Availability

Genbank Accession Numbers for sequences used in molecular phylogenetic analysis are listed in Table 5. Materials examined in this study and associated museum number are listed in Table 1.
